# Application of deep learning artificial intelligence technique to the classification of clinical orthodontic photos

**DOI:** 10.1186/s12903-022-02466-x

**Published:** 2022-10-25

**Authors:** Jiho Ryu, Yoo-Sun Lee, Seong-Pil Mo, Keunoh Lim, Seok-Ki Jung, Tae-Woo Kim

**Affiliations:** 1 Department of Orthodontics, School of Dentistry, Dental Research Institute, Seoul National University, 101 Daehakro, Jongro-gu, 03080 Seoul, Korea; 2 Department of Orthodontics, Korea University Guro Hospital, 148 Gurodong-ro, Guro-gu, 08308 Seoul, Korea

**Keywords:** Clinical photographs, Artificial intelligence, Deep learning, Orthodontics

## Abstract

**Background:**

Taking facial and intraoral clinical photos is one of the essential parts of orthodontic diagnosis and treatment planning. Among the diagnostic procedures, classification of the shuffled clinical photos with their orientations will be the initial step while it was not easy for a machine to classify photos with a variety of facial and dental situations. This article presents a convolutional neural networks (CNNs) deep learning technique to classify orthodontic clinical photos according to their orientations.

**Methods:**

To build an automated classification system, CNNs models of facial and intraoral categories were constructed, and the clinical photos that are routinely taken for orthodontic diagnosis were used to train the models with data augmentation. Prediction procedures were evaluated with separate photos whose purpose was only for prediction.

**Results:**

Overall, a 98.0% valid prediction rate resulted for both facial and intraoral photo classification. The highest prediction rate was 100% for facial lateral profile, intraoral upper, and lower photos.

**Conclusion:**

An artificial intelligence system that utilizes deep learning with proper training models can successfully classify orthodontic facial and intraoral photos automatically. This technique can be used for the first step of a fully automated orthodontic diagnostic system in the future.

## Background

The basic preparation for an orthodontic treatment plan comprises taking radiographic films such as cephalometric and posteroanterior films, impressions of a patient’s study stone model, and a series of clinical photos. Clinical intraoral and facial photos are useful for orthodontists and are essential [1, 2] in the initial diagnosis procedure. Intraoral photographs provide a variety of information about tooth shape, alignment, and gingival status. Facial photos provide esthetic features of facial shape and relationship with teeth. Clinical photos can be used independently to check the conveying clinical features, or they can be used with a combination of other diagnostic materials like stone models and radiographic image measurements.

Most studies on artificial intelligence related to orthodontics have focused on two [3–5] or three dimensional [6] digital radiograph films or numerical analyses with numbers which were already generated by humans [7, 8]. However, to the best of our knowledge, only few studies [9] to date have focused on digital orthodontic photographs. Thus, this study might be in the first group regarding artificial intelligence to classify orthodontic digital clinical photos. Among the future automated diagnostic steps, the automated orientation classification of facial and intraoral photos fed in a randomized order will be the first step to apply artificial intelligence into digital orthodontics.

As artificial intelligence has emerged to provide a new paradigm in the dental and medical fields, deep learning technique, a subset of artificial intelligence that uses convolutional neural networks (CNNs) system, has gained popularity in the area of graphic image analysis. Deep learning is a part of machine learning that is designed to mimic the recognition system of the human brain while harnessing computing power of graphic processing units [10, 11]. It utilizes artificial neurons that calculate weighted inputs to generate a single integrated output value by a simple classifier model that is similar to human pattern recognition [12]. To date, numerous studies on deep learning with medical images have already been published, and some techniques (i.e., simple classification of skin cancers by photos) are said to be as accurate as human experts [13, 14]. In the field of dentistry, studies on the automated detection of dental plaque [15] and radiographic cephalometric landmarks even up to 80 landmarks [16], have been published. Among the algorithms of deep learning, CNNs are reported to be commonly used [12] and well-suited for image processing including that of medical images [17, 18]. In particular, CNNs utilize a hierarchical structure for passing information about prominent features to following layers while exploiting the spatially local correlations between them [19].

The aim of this study was to build a CNNs model for the image type classification of clinical orthodontic photos one by one including four facial photos (front, smile, three-quarter, and right profile) and five intraoral photos (front, upper, lower, left buccal, and right buccal). Computerized training and validation of the model was performed, followed by testing the prediction accuracy.

## Methods

### Subjects

In this study, total 4448 clinical photos from 491 patients who visited the Seoul National University Dental Hospital for orthodontic treatment were included. There were 213 male and 278 female subjects, and the mean age was 21.3, ranging from 5 to 51. The photos were extracted from the database according to their categories. Only patient’s age and sex were obtained from the meta-data. The raw files were stored in a separate storage in a single workstation without any personally identifiable information. For the facial photos, the upper portion including the eye region was cropped to protect subject’s privacy.

### Photographic procedure

**Fig. 1:**
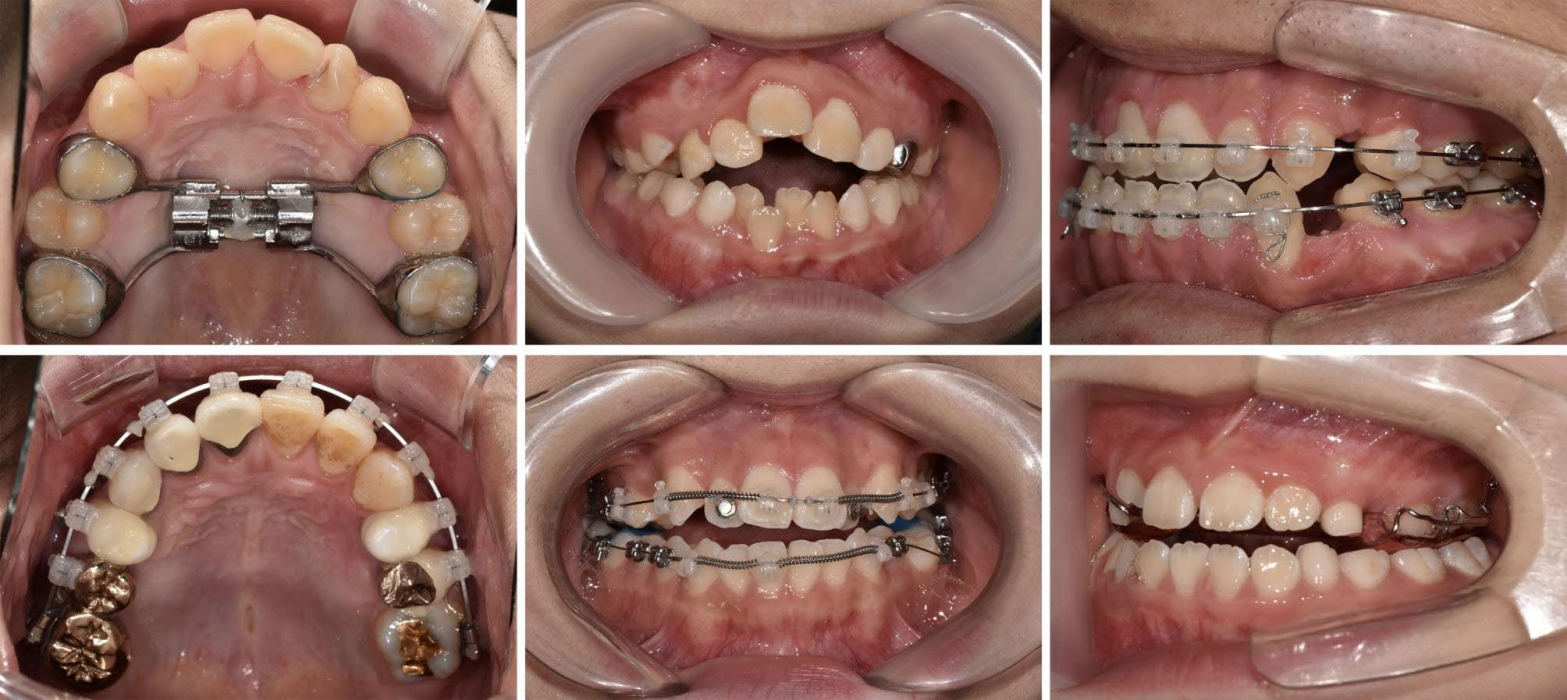
Examples of input photos. The different arrangements, alignments, appliances and statuses of teeth are shown

Several different doctors took the digital photos and there exists no reference about which doctor took each set of photos; in other words, different doctors could have taken photos of the same patient. This may give a randomization power of diversity in photo quality. Facial photos in a single set consist of front, front smile, right profile, and three-quarter profile. The intraoral photo sets contain front, left buccal, right buccal, maxillary occlusal, and mandibular occlusal views. All of these photos comprise the basic set of clinical photos taken in the Department of Orthodontics at Seoul National University Dental Hospital and include the recommended set of diagnostic orthodontic photography [20]. To encompass a variety of real clinical situations, dental conditions such as missing teeth, braces, removable appliances, or any kind of prosthesis were not excluded (Fig. 1).

### Deep learning system settings

For computational processing, Python 3.6.4 programming language was used in Microsoft Windows operating system. The deep learning model was constructed by using Keras 2.2.2 which utilizes Tensorflow-GPU 1.6.0 as a backend. An NVIDIA GeForce GTX1080 (8 GB RAM) with 16 GB system RAM workstation was used.

### Datasets

The gathered learning set consists of 1,396 facial photos and 2,152 intraoral photos, and the prediction set consists of 400 facial photos and 500 intraoral photos (Table 1). Data labeling was initially performed by J.R., then K.L. manually confirmed the labeled assets. There was no confliction. The original photos were randomly transformed to expand the number of photos numerically that a maximum six graphically random processed photos were generated from each original photo. The transformations involved one or more processes of translation, blurring, magnification, and rotation, yet the ranges were limited so as not to make it difficult for clinicians to distinguish them. In detail, a range from 0% to a maximum of 8% of shear, magnification, horizontal shift, vertical shift, and Gaussian blur with a radius of five pixels was applied. The validation set, consisted of individual photo data, was automatically and randomly divided from the total learning dataset without human intervention by using internal functions of Keras and Python Scikit-learn package. This splitting was done by each photograph object. The ratio of the training and validation sets was 75 to 25, which yielded 8,355 facial and 12,902 intraoral training images.

**Table 1:** Number of photos of each category

Category		Original	Augmented	Learning Set	Prediction Test Set
Facial	Front	322	2244	2566	100
	Front Smile	349	2435	2784	100
	Three-quarter	358	2497	2855	100
	Right Profile	367	2569	2936	100
	Mean	349	2436	2785	100
	Total	1396	9745	11,141	400
Intraoral	Upper	458	3206	3664	100
	Lower	451	3157	3608	100
	Right	415	2903	3318	100
	Front	371	2588	2959	100
	Left	457	3197	3654	100
	Mean	430	3010	3441	100
	Total	2152	15,051	17,203	500

**Fig. 2:**
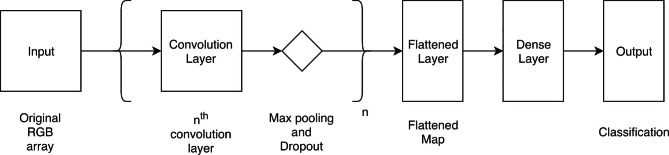
A basic network model structure consists of convolutional, pooling, dense, and dropout layers

### CNNs model architecture

Figure 2 depicts the overall CNNs model architecture for both facial and intraoral photo recognition. The training model is originally created which did not implement any pre-existing model. In the facial photo CNNs architecture, following the input layers, there are four convolution layers. All convolution layers follow with max pooling and dropout layers. Flatten, dense, dropout, and dense layers are present in sequence in the next-to-last convolution layer group. For the intraoral photo CNNs architecture, the layers are connected in the following order: single convolution, max pooling, dropout, flatten, dense, dropout, and another dense layer. The original data is put through a series of two-dimensional convolution layers with a stride parameter of three. All activation methods are rectified linear units algorithms (ReLU) [21]. The max pooling layer is then used to shrink the dimensions of the input layer that reduces the flowing data size. The two-dimensional 128-by-128-pixel input data is reduced to 64 by 64 pixels and then transformed through a flattened layer and categorized into four or five classifications with a Softmax activation layer according to the facial or intraoral input data type.

### Training and validation

Training the raw data sets were processed separately according to the facial and intraoral group. The Adam optimization method [22] and Categorical Cross-entropy loss function [18] were used. The training image data was fed to the model with a batch size of 32 and epoch of 50 cycles. After the training process was completed, the validation process was conducted with the rest of the learning image data, which were different from the training data.

### Prediction

The prediction procedure was performed with 100 clinical photos in each category individually, which were not duplicates from either the training or the validation sets. The prediction dataset contains intraoral photos with orthodontic appliances such as braces, screws, and transpalatal arches. There were 24 out of 100 patients having any intraoral orthodontic appliances in the prediction group. The testing photos were analyzed one by one as a single object, not as a set of patient case. The photos fed were neither arbitrarily flipped nor rotated. The predicted results were printed out as plain text with the label of the most likely photo category.

## Results

### Training and validation procedure

**Fig. 3:**
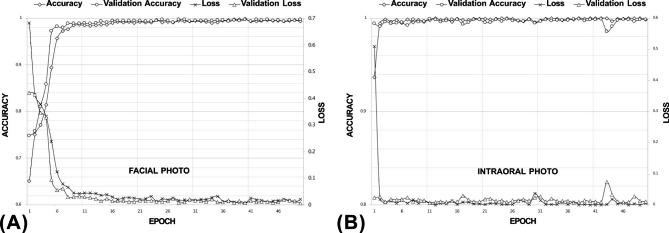
The learning curve of the training process. (**A**) Facial photo classification. (**B**) Intraoral photo classification

At the end of the training procedure, the training accuracy reached 99.3% for facial photos and 99.9% for intraoral photos (Fig. 3). The numbers of photos in the total validation set were 2,786 for facial photos and 4,301 for intraoral photos, which were randomly divided from the original mixed set. The validation accuracy values were 99.8% for both facial and intraoral photo classification.

### Prediction procedure

The prediction procedure was performed independently from the learning procedure, which means there were no duplicated photos for learning and prediction testing. Therefore, it is possible to evaluate the real situation of classifying never-trained new clinical photos. Every prediction test set consisted of photos from one hundred randomly picked patient cases that no patient case overlapped in the training dataset. The selection was made by patient case, not by photo object; hence the images were from one hundred different patients.

The mean rates of the successfully classified facial and intraoral photos were both 98.0%. Each successful classification rate is summarized in Table 2. In the classification of the facial photos, the highest success rate was 100.0% in detecting the right profile photos, and the lowest success rate was 97.0% in recognizing the front and smile photos. For the intraoral photos, the upper and lower photo detection rates were 100.0%, which were the highest, while the intraoral front photo had a detection rate of 94.0%, which was the lowest.

**Table 2:** Prediction test success rate. Total mean 98.0%

Category		Test Photos	Success Count	Success Rate (%)
Facial	Front	100	97	97
	Front Smile	100	97	97
	Three-quarter	100	98	98
	Right Profile	100	100	100
			Mean	98.0
Intraoral	Upper	100	100	100
	Lower	100	100	100
	Right	100	99	99
	Front	100	94	94
	Left	100	97	97
			Mean	98.0

## Discussion

There have been attempts to use machine learning methodology to help solve orthodontic problems like automated cephalometric landmarking and surgery versus non-surgery determination [8]. However, there had been a single study [9], to the best of our knowledge, to recognize and classify orthodontic clinical photos. In this study, we used a data augmentation technique with a lesser number of original photos compared to the previous study while maintaining a lightweight model structure. As labeling and annotating a large number of objects is a labor-intensive task, it would be a reasonable strategy to take a small number of samples for future practical implementation. This augmentation procedure was suitable for photo orientation classification because those photos are well standardized in general. In addition, the composition of patients’ photographs is also different from the previous article in that our dataset includes various conditions like orthodontic braces, screws, custom appliances, and combinations of them from any point during the treatment time.

Unlike a machine learning problem that calculates number data, problems regarding flat photos needs a different approach. To train a simple machine learning model to diagnose whether or not a patient needs a teeth extraction, for example, the operator may put previously calculated cephalometric analysis indices that are commonly used for deciding extractions, like an angle between A and B point (“ANB”), maxillary central incisor to Sella to Nasion line angle (“SN”), and upper lip to E-line, etc. [23]. However, deep learning technique work with two-dimensional chromatic photos composed of multitudinous pixels gathered together to make one single photo to be processed.

There will always be overfitting problems in the deep learning approach. Overfitting is generally expressed as good results on the training and validation sets but rather significantly decreased rates on the prediction set. This occurs when the model learns idiosyncratic features and memorizes parameters in more complicated patterns, which fit well to the training data, but fails to generalize the feature patterns [12]. To minimize this issue while improving the success rate, this training model uses the dropout technique to intentionally reduce the best-matching connection features, and augmentation of the input data as well [24]. For the augmentation, we implemented some degree of random transformation to inflate the sample photo number from 1,396 to 11,141 for facial photos and from 2,152 to 17,203 for intraoral photos.

**Fig. 4:**
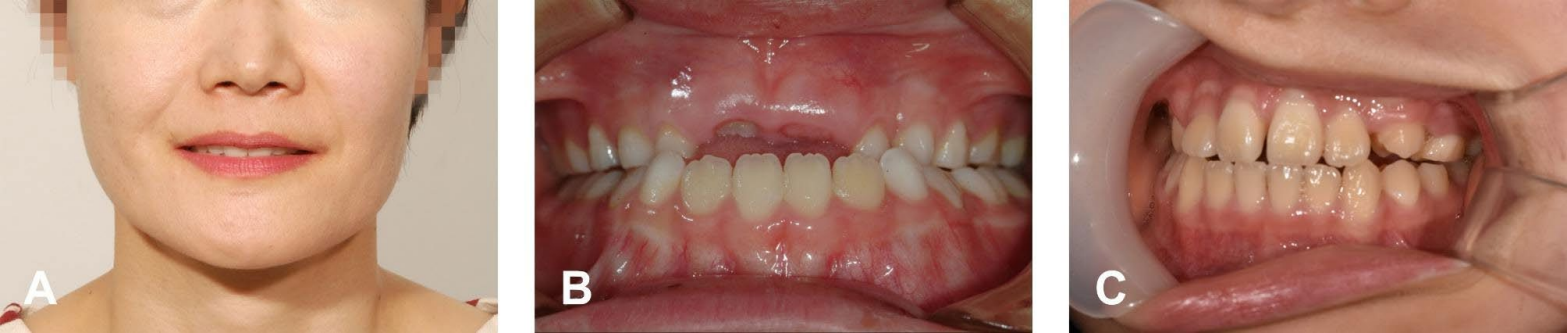
Examples of wrong predictions. (**A**) Facial smile photo that was classified as a front photo (cropped from the raw image). (**B**) Intraoral front photo that was classified as an intraoral right photo. (**C**) Intraoral left photo that was classified as an intraoral front photo

The reason for the relatively lower success rate in distinguishing the front facial and smile photos among the other facial photos may be attributed to the only minor difference between smiling and non-smiling, especially because we request patients to pose a light smile (Fig. 4A). Although the exact underlying mechanism of how the deep learning model makes its decisions has been barely known due to its nature [25], we assume that the model recognizes at least morphological differences like an outline of objects or color differences, lip contour, or white teeth exposure when smiling, which might not truly understand what ‘smiling’ means.

In addition, though still more than nine out of ten, the relatively lower resulting detection rate for front intraoral photos may be due to classification predisposition toward the left or right side, and due to the fact that the model may not explicitly distinguish the central incisors, which can be used for a reference vertical line. For example, in Fig. 4B, the unusual arrangement of anterior teeth images is sparsely fed so that it may reduce the probability of the correct prediction. A poorly taken clinical photo also could be one of the factors for prediction failure (Fig. 4C). However, the incorrectly predicted photos did not show a significant disposition of errors.

Most patients visiting orthodontic clinics have clinical photos taken at least once [26]. Unquestionably, clinical photos are key in diagnosing patients along with radiographs and study cast models [27], and in this decade, such diagnostic procedures are being replaced with digital processes. For example, using an intraoral scanner to acquire three-dimensional data with an automatically generated digital orthodontic study cast is equivalent to taking impressions. Taking three-dimensional cone-beam computed tomography to automatically extract lateral, posterior-anterior cephalograms, and even panoramic view is equivalent to taking each specific modality of radiograph. Automated segmentation and labeling of each tooth on radiograph images [28] and study casts [29] are being researched and applied in industries. Automated tracing and landmarking online services using artificial intelligence like WebCeph (AssembleCircle; Gyeonggi-do, South Korea) and CephX (ORCA Dental AI; Herzliya, Israel) are already being serviced. The purposes of these services are not only to save time for doing traditionally time-consuming procedures, but also to help improve human diagnosis by harnessing artificial intelligence.

Some limitations of this study are that the entire photos consisted of subjects from a single institution in South Korea, and the samples were manually preprocessed including cropping, resizing, and adjusting brightness and contrast by clinicians. To ensure further generalization of the prediction model, external validation with different institutions is needed [30]. Because CNNs process with color data, the colors of hair and skin and the general morphology of the face could affect the model. However, this can easily be overcome later by adding photos of other ethnic races because this deep learning model has the capability of learning regardless of varied inputs. In this study, for example, the learning model successfully detected photos with or without braces, with anterior teeth extracted, teeth malformations, removable appliances, etc. This could be done because the model learned the patterns of the data with key features that were also generated in the training process. Second, to avoid both complexity and divergency, and to get consistency and better explanations, we used the manually edited photos for real diagnosis situations that may influence the success rate [31]. However, such modification is not uncommon to orthodontists and may not be a limiting factor for the training data. Moreover, to overcome the variability of human interference, the photos were augmented with randomized preprocessing. In the same context, the datasets comprise clinical photographs taken in the department of orthodontics, which means there exist rather standardized formats like orientations and types, for example, consistently taking a patient’s right profile without a left profile. This can be regarded as a characteristic of orthodontic photo. As flipped asymmetric images put into the CNNs model may result in different outcomes [32], it needs a different model structure, dataset, and training strategy to make more generalized models that can distinguish those mirror images. Yet, in this study, we have narrowed the types of photos to be non-flipped ones both in the training and prediction processes, like in ordinary clinical circumstances.

This kind of two-dimensional deep learning classification study is only the first step in the field of automated orthodontic dentistry. In the future, deep learning artificial intelligence systems could be used for more diverse aspects of diagnoses by parameterizing clinical photos including molar and canine key detection, overjet and overbite estimation, etc. Moreover, like the three-dimensional automatic analysis of computerized tomography, it seems that automatic analysis of three-dimensional facial scans including treatment planning and soft tissue prediction would be possible someday. Altogether, better diagnosis and treatment planning can be accomplished in a more efficient and accurate manner for patients.

## Conclusions

Using a deep learning system with an artificial intelligence CNNs model, the facial and intraoral clinical photos that are routinely taken for orthodontic diagnostic purposes were automatically classified with an overall success rate of 98%. This study suggests that artificial intelligence can be applied to digital color photos to assist in the automation of the orthodontic diagnosis process.

## Data Availability

The datasets used and/or analyzed during the current study are available from the corresponding author on reasonable request.

## Abbreviations

CNNs: Convolutional neural networks.
ReLU: Rectified linear units algorithms.
